# Examining the impact of ADHD, pharmacological treatment, and internet addiction on the parent-adolescent relationships quality

**DOI:** 10.3389/fpsyg.2026.1761478

**Published:** 2026-05-01

**Authors:** Ahmet Özaslan, İrem Aktar Songür, Samet Can Demirci, Şeyma Gürbüz, Murat Yıldırım, Alican Kaya, Muhammed Celal Uras, Valentina Lucia La Rosa, Aydan Vehbi, Esra Güney, Elvan İşeri, Elena Commodari, Juan Gómez-Salgado

**Affiliations:** 1 Department of Child and Adolescent Psychiatry, Faculty of Medicine, Gazi University, Ankara, Türkiye; 2 Psychology Research Centre, Khazar University, Baku, Azerbaijan; 3 Department of Child and Adolescent Psychiatry, Lokman Hekim University, Ankara, Türkiye; 4 Department of Child and Adolescent Psychiatry, Etlik City Hospital, Ankara, Türkiye; 5 Department of Psychology, Faculty of Science and Letters, Ağrı İbrahim Çeçen University, Ağrı, Türkiye; 6 Department of Guidance and Psychological Counselling, Ağrı İbrahim Çeçen University, Ağrı, Türkiye; 7 Department of Elemenatary Mathematics Education, Ağrı İbrahim Çeçen University, Ağrı, Türkiye; 8 Department of Educational Sciences, University of Catania, Catania, Italy; 9 Department of Sociology, Social Work and Public Health, Faculty of Labour Sciences, University of Huelva, Huelva, Spain; 10 Safety and Health Postgraduate Program, Universidad Espíritu Santo, Guayaquil, Ecuador

**Keywords:** ADHD, adolescents, internet addiction, parent-adolescent relationship quality, treatment

## Abstract

**Introduction:**

Parent-adolescent relationship quality is vital for youth’s emotional and behavioral development; yet, it may be adversely impacted by neurodevelopmental disorders like attention-deficit hyperactivity disorder (ADHD). This study aimed to examine whether ADHD diagnosis and medication status are associated with differences in specific dimensions of parent–adolescent relationship quality – namely open communication, positive relationship quality, and negative relationship quality – while accounting for internet addiction as a contextual covariate.

**Methods:**

A total of 155 adolescents, aged 12–18 (*M* = 14.23 years, SD = 1.83), participated in this study. The participants were categorized into three groups: adolescents without ADHD (control group, *n* = 58), those with ADHD not receiving medication (*n* = 55), and adolescents with ADHD for at least 3 months (*n* = 42). We evaluated the parent-adolescent relationship quality across three dimensions: positive, open, and negative relationship quality with both parents. Multivariate Analyses of covariance (MANCOVA) were utilized to assess the impact of ADHD diagnosis and pharmacological treatment on relationship quality, controlling for age, sex, and internet addiction (IA) as factors.

**Results:**

After controlling for age and internet addiction, medication naïve adolescents with ADHD had significantly lower levels of open communication with mothers (*p* = 0.002) and fathers (*p* < 0.001) compared to adolescents with ADHD on medication and the control group, with the latter two groups not differing significantly from one another in this sample. No significant group differences were observed in negative relationship quality, and other positive relationship dimensions largely converged across groups. A higher level of IA was associated with higher negative relationship quality with both parents (*p* = 0.001) across the full sample; whether this association differs by ADHD diagnosis or medication status was not formally tested and cannot be determined from the present analyses. We identified age as a factor of lower father–adolescent relationship quality (*p* = 0.022). Still, we found sex to be insignificant (*p* > 0.05).

**Discussion:**

In conclusion, group differences were domain-specific: medication-naïve adolescents with ADHD reported significantly lower open communication with both parents, whereas no robust group differences emerged for positive or negative relationship quality. In contrast, adolescents receiving pharmacological treatment did not significantly differ from the control group on these dimensions in this sample. These findings suggest that pharmacological treatment status is associated with differences in open communication patterns in families with adolescents with ADHD; however, the cross-sectional and non-randomized nature of our design precludes causal inference. Also, the finding that IA showed a significant main effect on relationship quality across the full sample highlights the importance of considering internet usage patterns when working with families of adolescents with ADHD.

## 1 Introduction

Attention-deficit/hyperactivity disorder (ADHD) is one of the most common neurodevelopmental conditions in children and adolescents. It affects an estimated 7.6% of children under 12 and 5.6% of 12–18-year-olds (Salari et al., 2023). Beyond its well-documented cognitive and behavioral consequences, including inattention, impulsivity, emotional dysregulation, and comorbid psychopathology, ADHD exerts a pervasive influence on the social ecology of development.

One of the most consistently affected domains is the parent–adolescent relationship (Giannakopoulos, 2025; Miller et al., 2008; Ogundele and Ayyash, 2023). Developmental research has long recognized that family relationships and child behavioral characteristics influence one another over time; within this broad perspective, early parenting characteristics have been prospectively linked to ADHD severity (Ellis and Nigg, 2009; Thorell et al., 2012), and poorer relationship quality associated withoppositional defiant disorder symptoms (Bohlin et al., 2012; Wymbs et al., 2015). Although the present cross-sectional design cannot test these dynamic, longitudinal processes, it is well-suited to examine whether ADHD diagnostic status and medication status are associated with differences in specific relational dimensions at a given point in time — and, if so, which dimensions are most sensitive to these clinical variables.

Adolescence intensifies the demands on these relationships. As adolescents seek autonomy, form identities, and renegotiate authority, the relational context becomes particularly consequential for psychosocial adjustment (Garcia et al., 2019). Frustration intolerance, poor conflict resolution skills, and diminished responsiveness are well-documented features of ADHD during this developmental period and among the most impaired interpersonal competencies in this population (Garcia et al., 2019; Markel and Wiener, 2014). Crucially, these behavioral features are not equally likely to affect all dimensions of relationship quality. Open communication — which captures moment-to-moment disclosure, reciprocal exchange, and responsiveness to parental cues — is continuously exposed to attentional and regulatory demands and therefore more proximally sensitive to current symptom status than positive or negative relationship quality, both of which reflect more consolidated affective appraisals shaped by accumulated relational history (Kapetanovic et al., 2019).

However, empirically, the picture is more complex. Common conceptualizations of the quality of the parent-adolescent relationship distinguish three dimensions: open communication (disclosure, reciprocity, and responsiveness); positive quality (proximity, warmth, and satisfaction); and negative quality (conflict and antagonism) (Dekovic et al., 2003; Wissink et al., 2006). Studies on adolescents indicate that greater ADHD symptom severity predicts poorer relationships with mothers and that symptom severity and relational processes are bidirectionally associated over time (Fleck et al., 2015; Lifford et al., 2008). However, findings across specific relational dimensions are inconsistent due to variability in samples, measures, and the extent to which comorbid conditions and contextual factors are controlled (Lange et al., 2005; Sciberras et al., 2014).

Importantly, two contextual factors that may substantially shape the association between ADHD and relationship quality have rarely been examined simultaneously: pharmacological treatment status and internet addiction (IA). Standard ADHD management includes pharmacological and non-pharmacological treatment strategies that reduce core symptoms and improve functional outcomes and quality of life (Bellato et al., 2025; Cannon et al., 2009). Some evidence suggests that stimulant medication improves parent–child communication and child compliance and reduces negative maternal responses (Barkley et al., 1992; Pelham et al., 2017). However, evidence specific to the parent–adolescent relationship is mixed. Some studies report no relational effects of stimulants despite symptom improvement (Pelham et al., 2017). Whether medication status is associated with differences across specific relational dimensions and whether these associations are domain-specific or global remains an open question of direct clinical relevance. Practice guidelines identify pharmacological treatment as a recommended component of ADHD management, in part, on the basis of its potential relational benefits (Wolraich et al., 2019). Furthermore, adolescents with ADHD are substantially more likely than their typically developing peers to develop problematic internet use, possibly via shared mechanisms of rewards sensitivity and executive control deficits (Enagandula et al., 2018; Ko et al., 2009). IA is associated with parent–adolescent conflict, decreased relational warmth, and inconsistent monitoring (Ding et al., 2017; Lukavska et al., 2020; Yen et al., 2007). In turn, parent–adolescent conflict has been linked to subsequent IA onset (Zhou et al., 2017). Since IA is a likely correlate of ADHD and an independent predictor of relationship quality, failing to model IA as a covariate can inflate or obscure the estimated associations between ADHD status and relational outcomes. This methodological concern has rarely been addressed in prior studies in this area.

Taken together, these considerations reveal a clear gap in the literature: no study has compared the quality of parent-adolescent relationships across groups with and without an ADHD diagnosis while accounting for IA as a contextual covariate. Additionally, no prior work has examined whether medication status is associated with differences in specific relational dimensions rather than global relationship quality. The present study directly addresses this gap. Using a three-group, cross-sectional design (medication-naïve adolescents with ADHD, medicated adolescents with ADHD, and typically developing controls), and modeling six relational outcomes across three dimensions and two parental figures with IA as a covariate, we examined whether parent–adolescent relationship quality differs by ADHD diagnosis and medication status in domain-specific or global ways and whether IA accounts for unique variance in relational outcomes beyond group membership. This design has direct clinical relevance. Identifying which relational domains are most sensitive to ADHD symptoms and their pharmacological management can inform intervention priorities for families with adolescents who have ADHD.

Based on previous literature, we formulated the following hypotheses. First, based on evidence linking ADHD symptoms to reduced communication (Barkley et al., 1992; Garcia et al., 2019), and consistent with the theoretical rationale outlined above, we predicted that medication-naïve adolescents with ADHD would report lower levels of open communication with both parents compared to typically developing controls. We did not make directional predictions for positive or negative relationship quality, given the mixed and less consistent findings for these dimensions in prior literature. Additionally, we predicted that medicated adolescents with ADHD would report higher levels of open communication than medication-naïve adolescents. Second, based on evidence linking IA to family conflict and reduced relational warmth (Ding et al., 2017; Zhu et al., 2022), we predicted that higher IA scores would be associated with poorer relationship quality across the full sample. We also examined the group × sex interaction as an exploratory term, motivated by prior evidence of differential parental responsiveness by adolescent sex (Chronis-Tuscano et al., 2008), but we did not make a directional prediction.

## 2 Materials and methods

### 2.1 Participants

This cross-sectional observational study was conducted between December 2022 and December 2025 at the Child and Adolescent Psychiatry Outpatient Clinic of Gazi University Faculty of Medicine. Participants were consecutively recruited among adolescents aged 12–18 years who presented to the clinic during the study period. A consecutive sampling method was employed to minimize potential selection bias. Diagnostic assessments were conducted by a trained child and adolescent psychiatrist (AÖ). Pre-screening of consecutive outpatient attendees for broad eligibility was performed by research assistants (ŞG, İAS, SCD). The ADHD group consisted of adolescents meeting DSM-5 diagnostic criteria for Attention-Deficit/Hyperactivity Disorder, either through a prior diagnosis or one newly established during the study evaluation. The inclusion criteria were determined as having no psychiatric diagnosis other than ADHD during the past year and having no known chronic medical condition, based on the assessment conducted with the *Schedule for Affective Disorders and Schizophrenia for School-Age Children—Present and Lifetime Version—DSM-5—Turkish Version (K-SADS-PL-DSM-5-T)*. The case group was divided into two subgroups according to medication status: Adolescents diagnosed with ADHD who were not receiving medication (medication-naïve ADHD; MNAD), and adolescents with ADHD who had been receiving pharmacological treatment for at least 3 months before participation (medicated ADHD; MEDAD). For adolescents in the MEDAD group, information on current pharmacological treatment was obtained from medical records and parental report, including medication class, daily dose for ADHD medication, and the presence of additional psychotropic medication use. This allowed descriptive characterization of the medicated group in terms of stimulant versus non-stimulant treatment and monotherapy versus combination treatment. However, detailed information on treatment duration beyond the 3-month threshold, stable dose status, treatment adherence, side effects, clinical response, and antipsychotic dose was not systematically available. Participants were excluded if they: (1) declined to participate after being informed about study procedures; (2) met DSM-5 criteria for any comorbid psychiatric disorder other than ADHD, as identified through the K-SADS-PL-DSM-5-T; or (3) had a known chronic medical condition. The control group was recruited separately during the same study period from adolescents in the same age range who did not meet criteria for ADHD or any other psychiatric disorder within the past year. Controls were recruited through convenience sampling from adolescents who were accessible during the study period and met eligibility criteria. Control participants were required to have no known chronic medical condition and were screened using both clinical evaluation and the K-SADS-PL-DSM-5-T. No formal individual matching procedure was used; instead, the control group was selected to be broadly comparable to the ADHD groups in terms of age and sex distribution.

### 2.2 Measures

A sociodemographic data form, developed by the research team and completed by parents, collected information on adolescents’ age, sex, grade level, medication status, and predominant ADHD presentation.

To assess ADHD symptomatology, parents completed the *Revised Conners’ Parent Rating Scale* (CPRS-RS), a 27-item measure rated on a four-point Likert scale (0 = never to 3 = always) that yields three subscales - Oppositional, Cognitive Problems-Inattention, and Hyperactivity - along with an ADHD Index. Higher scores reflect greater symptom severity, and the Turkish version demonstrates good internal consistency (α = 0.73–0.91) (Kaner et al., 2013). In the current sample, internal consistency coefficients were also acceptable (α = 0.78–0.91).

Parent–adolescent relationship quality was assessed using the *Parent-Adolescent Relationship Quality Scale* (PARQ), an 18-item instrument evaluating descriptive, positive, and negative dimensions of the relationship. Items are rated separately for mothers and fathers on a five-point Likert scale (1 = not at all to 5 = very much) (Wissink et al., 2006), with the Turkish adaptation showing strong reliability (α = 0.80–0.88) (Batıgün and Say, 2015). In the current sample, internal consistency coefficients were also acceptable (α = 0.78–0.86). Adolescents also completed *Young’s Internet Addiction Test* (YIAT), a 20-item measure developed by Young (1998) and adapted into Turkish by Bayraktar (2001), scored from 0 (not applicable) to 5 (always), yielding total scores from 0 to 100. Higher scores indicate more severe IA, and the Turkish version demonstrates excellent reliability (Bayraktar and Gun, 2007). In the current sample, internal consistency coefficients were also acceptable (α = 0.89).

Finally, psychiatric diagnoses were evaluated using the *Schedule for Affective Disorders and Schizophrenia for School-Age Children- Present and Lifetime Version, DSM-5, Turkish adaptation* (K-SADS-PL-DSM-5-T). First developed by Kaufman et al. (1997) and translated to Turkish by Unal et al. (2019), this semi-structured diagnostic interview, administered by trained child psychiatrists, integrates parent and adolescent reports with clinical observation (Kaufman et al., 1997; Unal et al., 2019). It was used to confirm ADHD diagnoses and to ensure the absence of psychiatric comorbidities in accordance with the study’s inclusion criteria. As a semi-structured diagnostic interview, the K-SADS-PL-DSM-5-T does not yield a continuous total score; accordingly, internal consistency coefficients are not applicable for this instrument. Reliability is established through inter-rater and test-retest procedures, as reported in the Turkish adaptation (Unal et al., 2019).

### 2.3 Procedure

This study was conducted and reported in accordance with the STROBE (Strengthening the Reporting of Observational Studies in Epidemiology) guidelines for cross-sectional studies. Adolescents who completed routine outpatient evaluations and met the inclusion criteria were invited to participate. Study aims and procedures were explained to adolescents and their parents, who provided written informed consent before participation. All assessments were administered individually in a quiet, private room. The K-SADS-PL-DSM-5-T interviews were conducted exclusively by a single trained child and adolescent psychiatrist (AÖ), who had no access to participants’ medication status, group assignment, or prior diagnostic history at the time of assessment. Pre-screening of consecutive outpatient attendees for broad eligibility was performed by research assistants (ŞG, İAS, SCD), who were aware of participants’ clinical background and medication status. Group allocation was determined only after the blinded K-SADS interview was completed. Subsequently, adolescents completed the PARQ and YIAT under researcher supervision, while parents completed the Sociodemographic Data Form and CPRS-RS.

### 2.4 Statistical analysis

Given the lack of an *a priori* power estimation, we conducted a *post hoc* sensitivity analysis using G*Power 3.1. The analysis showed that with α = 0.05, 80% power, and a total sample of 155 across three groups, the minimum effect size detectable by our design was f = 0.25, corresponding to a small-to-medium magnitude. All analyses were performed using IBM SPSS Statistics 29.0 (IBM Corp., Armonk, NY, United States), with significance set at *p* < 0.05 (two-tailed).

Group differences in parent–adolescent relationship quality were examined using MANCOVA with six dependent variables (open, positive, and negative relationship quality rated separately for mother and father), two fixed factors (group: control, MNAD, MEDAD; sex: male, female), and two covariates (age and YIAT internet addiction score). The group × sex interaction term was included in all models as an exploratory term. Covariates were selected *a priori* based on theoretical grounds: age was included given normative developmental changes in parent–adolescent relationships across adolescence, and internet addiction was included given established associations with family conflict and its elevated prevalence in ADHD samples. Pillai’s Trace was used as the multivariate test statistic given the significant Box’s M test (*p* < 0.001). Follow-up univariate ANCOVAs with Bonferroni-adjusted pairwise comparisons were conducted for each of the six relationship dimensions to control Type I error. Model assumptions (normality, linearity, and homogeneity of variances/covariances) were checked and satisfied. Partial η^2^ values were reported as effect sizes.

## 3 Results

In the control group, 17 adolescents declined participation and five were excluded following K-SADS-PL-DSM-5-T assessment due to identification of a psychiatric disorder, resulting in a final sample of 58. In the medication-naïve ADHD group, 13 adolescents were excluded due to psychiatric comorbidity, seven declined participation, four had incomplete forms, and one had a chronic medical condition, resulting in a final sample of 55. In the medicated ADHD group, five were excluded due to psychiatric comorbidity, three declined participation, two had incomplete forms, and one had a chronic medical condition, resulting in a final sample of 42. Thus, a total of 155 adolescents were included in the final analysis. As shown in Table 1, the sample comprised three groups of adolescents: a control group without ADHD (*n* = 58), adolescents with ADHD who had not received medication (medication naïve-ADHD; MNAD; *n* = 55), and adolescents with ADHD who had been on pharmacologic treatment for a minimum of 3 months before participation (medicated ADHD; MEDAD; *n* = 42). Within the MEDAD group (*n* = 42), 10 adolescents were receiving atomoxetine and 32 were receiving methylphenidate. Atomoxetine doses ranged from 50 to 90 mg/day (median = 60 mg/day), and methylphenidate doses ranged from 18 to 54 mg/day (median = 27 mg/day). Of the 10 adolescents receiving atomoxetine, three were also receiving methylphenidate. Of the 32 adolescents receiving methylphenidate, six were also receiving risperidone or aripiprazole. Overall, 33 adolescents were receiving monotherapy and nine were receiving combination pharmacotherapy. The mean age of the adolescents included in the sample was 14.23 (SD = 1.83), with a range of 12–18. A one-way analysis of variance (ANOVA) indicated no significant group differences in mean age, *F*(2, 152) = 2.30, *p* = 0.103. Sex distribution was also balanced across groups (female = 59, male = 96), χ^2^ (2, *N* = 155) = 0.16, *p* = 0.923).

**TABLE 1:** Demographic characteristics of attention-deficit hyperactivity disorder (ADHD) and control groups.

Characteristic	MEDAD group (*n* = 42)	MNAD group (*n* = 55)	Control group (*n* = 58)	*P*-value
Age	14.19 ± 1.94	14.53 ± 1.41	13.98 ± 2.06	0.28
Sex				
Boys (%)	27	34	35	0.92
Girls (%)	15	21	27	–
ADHD presentation				
Inattentive, *n* (%)	22 (52.4)	30 (54.5)	–	–
Hyperactive-impulsive, *n* (%)	2 (4.8)	–	–	–
Combined, *n* (%)	18 (42.8)	25 (45.5)	–	–

The overall multivariate analysis of covariance (MANCOVA) revealed a significant multivariate main effect of group on the combined dependent variables of parent-adolescent relationship quality (see Table 2), Pillai’s Trace = 0.185, *F*(12, 286) = 2.43, *p* = 0.005, η^2^ = 0.092. This finding indicates that the overall pattern of relationship dimensions differed significantly across the three groups after adjusting for age and IA. In addition, there were significant multivariate effects of age (Pillai’s Trace = 0.098, *p* = 0.022) and IA (Pillai’s Trace = 0.204, *p* < 0.001), whereas no significant multivariate effects were found for sex (*p* = 0.364) or the group × sex interaction (*p* = 0.699). Regarding the covariates (see Table 2 for multivariate effects), internet addiction showed strong positive associations with negative relationship quality with both parents [*F*(1,147) = 28.17, *p* < 0.001, η^2^ = 0.161; F(1,147) = 24.19, *p* < 0.001, η^2^ = 0.141 for fathers], indicating that higher levels of internet addiction were linked to more conflictual relationships. Age was inversely associated with a positive relationship with fathers [F(1,147) = 8.31, *p* = 0.005, η^2^ = 0.05], suggesting slightly less positive relationships among older adolescents.

**TABLE 2:** Multivariate tests (Pillai’s Trace).

Effect	Pillai’s Trace	*F*	Hypothesis df	Error df	*P*	Partial η^2^
Group (3 levels)	0.185	2.43	12	286	0.005	0.092
Age (covariate)	0.098	2.56	6	142	0.022	0.098
Internet addiction (covariate)	0.204	6.08	6	142	<0.001	0.204
Sex	0.045	1.10	6	142	0.364	0.045
Group × sex	0.061	0.75	12	286	0.699	0.031

Pillai’s Trace values are reported due to the significant Box’s M test (*p* < 0.001).

Follow-up univariate ANCOVAs, summarized in Table 3, identified which specific relationship dimensions contributed to the overall multivariate effect. After controlling for age and IA, significant group differences emerged for open communication with mothers [F(2,147) = 6.74, *p* = 0.002, η^2^ = 0.08] and open communication with fathers [F(2,147) = 12.53, *p* < 0.001, η^2^ = 0.15]. Bonferroni-adjusted pairwise comparisons indicated that the medication-naïve ADHD group reported significantly lower open communication scores than both the control and medicated ADHD groups (all ps ≤ 0.007), whereas the medicated ADHD group did not significantly differ from the control group on these dimensions in this sample. A smaller group effect was observed for positive relationship quality with fathers [F(2,147) = 4.45, *p* = 0.013, η^2^ = 0.057], but this effect did not remain significant after applying the Holm-Bonferroni correction across the six univariate tests. Group differences in positive relationship with mothers and in negative relationship quality with either parent were non-significant (all ps ≥ 0.05).

**TABLE 3:** Univariate tests of group effects.

Dependent variable	MNAD mean (SE)	MEDAD mean (SE)	Control mean (SE)	*F*(2,147)	*P*	Partial η^2^
Open communication (mother)	17.04 (0.78)	20.44 (0.75)	20.76 (0.90)	6.64	0.002	0.083
Open communication (father)	13.79 (0.79)	18.94 (0.76)	18.45 (0.91)	12.68	<0.001	0.147
Positive relationship (mother)	17.09 (0.84)	19.49 (0.81)	19.69 (0.97)	2.83	0.062	0.037
Positive relationship (father)	14.75 (0.74)	17.81 (0.71)	16.72 (0.86)	4.45	0.013	0.057
Negative relationship (mother)	14.30 (0.75)	14.37 (0.72)	13.14 (0.86)	0.71	0.492	0.010
Negative relationship (father)	14.86 (0.74)	12.89 (0.71)	12.58 (0.85)	2.64	0.075	0.035

Covariates included age and internet addiction scores. Bonferroni-adjusted comparisons were used. Levene’s tests indicated potential variance inequality for positive relationship variables (*p* < 0.05).

## 4 Discussion

The present study compared parent-adolescent relationship quality across three groups of adolescents — those with ADHD not receiving pharmacological treatment, those with ADHD receiving pharmacological treatment for at least 3 months, and typically developing controls — while adjusting for age, sex, and IA as covariates. The central finding was domain-specific: medication-naïve adolescents with ADHD reported significantly lower open communication with both mothers and fathers compared with typically developing controls and with medicated adolescents with ADHD, whereas the latter two groups did not significantly differ from one another on these dimensions in this sample. Group differences did not extend to positive or negative relationship quality: a small effect for positive relationship quality with fathers emerged but did not survive correction for multiple comparisons, and no group differences were detected for negative relationship quality or for positive relationship quality with mothers. Across the full sample, IA showed a significant main effect on negative relationship quality with both parents, and older age was associated with slightly less positive relationship quality with fathers. Sex and group-by-sex effects were not significant. All group comparisons reflect associations with medication status and cannot be interpreted as evidence of treatment effects, given the non-randomized, cross-sectional design.

That group differences were confined primarily to open communication — rather than distributed across all three relational dimensions — is itself a substantively meaningful finding. Open communication reflects moment-to-moment interactional processes that are proximally sensitive to current symptom status, whereas positive and negative relationship quality capture more consolidated affective orientations less likely to vary with behavioral fluctuations in the present (Villarreal and Nelson, 2022). The lower level of openness observed in the medication-naïve ADHD group is consistent with prior evidence that core behavioral features of ADHD, including inattention and restlessness, have been associated with adolescent non-compliance and diminished responsiveness, which in turn have been linked to more controlling parental behaviors and reduced relational receptivity (Barkley et al., 1992). Speculatively, ADHD-related executive and emotional regulation difficulties may be associated with coercive interactional patterns that constrain open communication (Rahimi and Shojaei, 2017; Breaux and Langberg, 2020). However, this mechanism was not directly observed or tested in the present study. The pattern of attenuated group differences among adolescents receiving pharmacological treatment is consistent with prior reports suggesting that medication status is associated with improvements in daily functioning and relational processes (Lifford et al., 2008; Sibley et al., 2022), and may plausibly reflect improvements in attention and impulse control that support more open communicative exchange (Blader, 2021; Lu et al., 2019). However, all such interpretations are speculative, given that treatment response and interactional processes were not directly measured. This interpretation also aligns with recent evidence suggesting that links between ADHD and risky internet use are intertwined with social skills and interpersonal functioning (Derin et al., 2025).

At least three explanations for the null findings on positive and negative dimensions deserve consideration. First, IA showed particularly strong associations with negative relationship quality in the present sample, with effect sizes substantially larger than those observed for group differences on open communication. By accounting for a large portion of the variance in negative relationship quality as a covariate, IA may have reduced the residual variance available to detect group differences on that dimension — a pattern consistent with prior evidence that digital media use and family conflict are tightly coupled in adolescent samples ( Özaslan et al., 2021; Zhu et al., 2022). Had IA not been modeled, group effects on negative relationship quality might have been more apparent, though confounded. This nuance is noteworthy because the determinants of heightened conflict in families of adolescents with ADHD have previously been conceptualized broadly, encompassing academic underachievement, executive difficulties, and challenges with time management (Markel and Wiener, 2014); the present findings suggest that digital media use may represent an additional, undermodeled source of relational strain in this population. Second, differential statistical power across the six outcomes cannot be excluded: open communication yielded the largest group effect sizes in the sample, while effects on other dimensions were smaller and may have been undetectable given the present sample size. Replication in larger samples is needed to clarify whether these null findings are robust. Third, paternal involvement and sensitivity tend to be more variable in response to children’s behavioral difficulties than maternal warmth, which is relatively stable across contexts (Chronis-Tuscano et al., 2008). This may partly explain why the small positive relationship effect for fathers did not survive correction for multiple comparisons while maternal effects were uniformly non-significant. Taken together, the null findings on positive and negative dimensions reflect a combination of construct-level, analytic, and power-related factors, and should not be interpreted as evidence that these dimensions are unaffected by ADHD or medication status in the population.

A related but distinct consideration concerns the developmental context in which these null findings emerge. The absence of group differences on negative relationship quality contrasts with prior studies that have documented elevated conflict in families of adolescents with ADHD. Beyond the IA saturation argument outlined above, it is possible that unmodeled digital-media variables contributed to earlier ADHD–control discrepancies in the literature, though this remains speculative and cannot be formally evaluated within the present design. From a developmental perspective, normative processes during adolescence — including increasing autonomy seeking, renegotiation of authority, and age-graded fluctuations in relational appraisals — may have attenuated detectable differences between clinical and control groups, particularly given the wide age range of the sample (De Goede et al., 2009; Hadiwijaya et al., 2017; Mastrotheodoros et al., 2020). Parents and adolescents are also known to differ in their perceptions of conflict intensity, especially within father–adolescent dyads, with perceptual discrepancies shaped by individual personality traits (Branje, 2018; Mastrotheodoros et al., 2020; Nelemans et al., 2016), and such discrepancies may have further obscured group-level differences. The age-related decline in positive father–adolescent relationship quality observed across all groups is consistent with this developmental account: as parental and adolescent roles are renegotiated in the pursuit of autonomy, warmth and open communication may temporarily weaken in early to middle adolescence before partial recovery in later years, and individual variation in these trajectories reflects natural developmental reorganization rather than a uniform pattern (Branje, 2018; Hadiwijaya et al., 2017; Mastrotheodoros et al., 2020).

The exclusion of adolescents with comorbid psychiatric disorders, while enhancing internal validity, also warrants consideration as a factor shaping the pattern of findings. Parent-child interaction difficulties are closely linked to depression in adolescents with ADHD (Garcia et al., 2019), and parent–adolescent conflict has been shown to mediate the association between ADHD and depressive symptoms in both cross-sectional and longitudinal work (Eadeh et al., 2017; Humphreys et al., 2013). Targeting relational deficits in ADHD treatment during adolescence may therefore provide additional benefit for reducing risk for comorbid depression (Ward et al., 2019). The exclusion of comorbid cases likely removed a subgroup in which relational difficulties are particularly pronounced, thereby attenuating potential between-group differences. From a developmental-psychopathological perspective, suboptimal relationship quality may not be a core feature of ADHD *per se* but may become most evident when ADHD is accompanied by internalizing difficulties (Meinzer et al., 2021). The relatively favorable relational profiles observed in this study may thus reflect a subgroup of adolescents with ADHD who maintain adaptive relational dynamics in the absence of significant emotional comorbidity.

The significant association between IA and negative relationship quality across the full sample is consistent with a growing literature documenting bidirectional associations between ADHD symptom dimensions and problematic digital engagement across adolescence (Morita et al., 2022; Wang J.-L. et al., 2024), and between parent-adolescent conflict and subsequent IA onset at the familial level (Ko et al., 2015). Derin et al. (2026) further reported that parent-child conflict significantly explained the association between ADHD and risky internet use, underscoring the relevance of relational processes in interpreting IA-related findings in this population. A meta-analysis showed that poorer parent–child relationships are associated with higher IA scores cross-culturally (You et al., 2025), and evidence does not consistently indicate that parental roles moderate this relationship, aligning with the comparable effect sizes observed for mothers and fathers in the present study (Zhu et al., 2022). Derin et al. (2023) further showed that IA in adolescents with ADHD is associated with parental depression and anxiety, suggesting that parental mental health represents an important contextual factor not assessed in the present study. Whether the bidirectionality documented in longitudinal work characterizes the present sample cannot be inferred from the cross-sectional design, and because both IA and relationship quality were assessed via adolescent self-report, the observed association may have been partly amplified by shared reporter effects such as negative affectivity, response style, or generalized dissatisfaction. Regarding the relationship between pharmacological treatment and IA specifically, evidence is mixed, as some studies suggest that ADHD medication may reduce specific maladaptive technological behaviors such as excessive online gaming (Han et al., 2009; Park et al., 2016), while prospective findings indicate that methylphenidate may not have a distinct direct effect on IA trajectories when sociodemographic and clinical covariates are accounted for Salvati et al. (2024). Problematic internet use in adolescents with ADHD likely arises from cross-domain mechanisms, including emotional regulation and parental monitoring practices (Ding et al., 2017), rather than being exclusively linked to medication effects.

### 4.1 Strengths and limitations

This study has some important strengths. First, the evaluation of both medicated and unmedicated adolescents in the ADHD group allowed for the examination of intra-group clinical differences rather than treating ADHD as a uniform clinical group. Second, the separate evaluation of the relationship dimensions with the mother and father provided a more detailed and differentiated view of the parent-adolescent relationship quality. Third, the inclusion of internet addiction as a covariate in the analysis ensured that a current and clinically important variable that could affect parent-adolescent relationship processes in ADHD was taken into account. When these features are considered together, they strengthen the clinical significance of the study and the interpretability of the findings.

However, some limitations merit consideration. The cross-sectional design precludes causal inference regarding the directionality among ADHD, IA, and parent–adolescent relationship quality. Relationship quality relied on adolescent self-report without observational or multi-informant corroboration, raising potential shared-method variance. This is particularly important for the association between IA and relationship negativity, which may have been partly inflated by shared reporter effects such as negative affectivity, response style, or generalized dissatisfaction. Medication status was not randomized, and heterogeneity in pharmacological treatment type, dose, and duration was not fully characterized; thus, the “medicated” category may index differences in treatment exposure and broader clinical context. Although medication class, ADHD medication dose, and the presence of additional psychotropic medication use were available, detailed information on treatment duration beyond the minimum 3-month threshold, stable dose status, adherence, side effects, treatment response, and antipsychotic dose was not systematically available. Accordingly, the medicated group should be interpreted as a heterogeneous clinical category rather than a uniform treatment condition. Furthermore, no systematic data were collected on non-pharmacological interventions — including psychotherapy, behavioral parent training, school accommodations, or counseling — across any of the three groups. Accordingly, observed group differences should be interpreted as reflecting pharmacological treatment status rather than treatment in a broader sense, and the potential contribution of unmeasured psychosocial interventions cannot be excluded. Also, particular characteristics of the medication-naïve participants—such as the time since diagnosis, parental attitudes toward pharmacotherapy, and symptom severity—may systematically differ from those of the medicated group, potentially introducing selection bias. Furthermore, although ADHD presentation data were available, subgroup sizes were insufficient to examine presentation type as a moderator of parent–adolescent relationship outcomes; this remains an important direction for future research. Exclusion of psychiatric comorbidity enhances internal validity but may limit generalizability to typical outpatient populations. Additionally, family-level sociodemographic and socioeconomic variables — including parental education, marital status, household income, parental age, and employment status — were not systematically collected. Although the three groups were broadly comparable on age and sex distribution, residual confounding by these unmeasured variables cannot be excluded, and future studies should collect and report a fuller set of family-level covariates. Parental mental health and family-process variables such as parent-child conflict were also not measured, although recent studies suggest that these factors may be relevant contextual correlates of IA in adolescents with ADHD (Derin et al., 2023, 2026).

### 4.2 Clinical and research implications

The present findings carry several implications for clinical practice and future research, though these should be understood as tentative given the cross-sectional design and the limitations described in the following section. The domain specificity of the results suggests that open communication represents a particularly sensitive target for clinical assessment in families of adolescents with ADHD. Given its behavioral and interactional character, it may be more responsive to change than the more consolidated affective dimensions of relationship quality, and its disruption may have downstream consequences for treatment engagement and psychosocial adjustment. Despite the normative increase in parent–adolescent conflict during adolescence, open communication can be maintained, and family-focused approaches are well-positioned to support it (Branje, 2018; De Goede et al., 2009; Skinner et al., 2021). Communication-focused modules within behavioral parent training programs, which have demonstrated efficacy in improving parent–child relationship quality and parental self-efficacy in ADHD samples (Dekkers et al., 2022; Dekovic et al., 2003), may therefore represent a clinically meaningful adjunct to pharmacological management. Pharmacotherapy remains central to symptom control (Cortese et al., 2018), but evidence from the Multimodal Treatment Study of Children with ADHD indicates that lasting gains in family functioning are more likely when behavioral components complement medication-based strategies: at 14 and 24 months, medication-based approaches showed symptom superiority, yet significant difficulties in school, social, and family functioning persisted over an 8-year follow-up, suggesting that pharmacotherapy alone may yield limited relational gains (MTA Cooperative Group, 1999, 2004). The independent association between IA and relational negativity further suggests that digital media habits should be routinely assessed in clinical evaluations of adolescents with ADHD, and that family-based interventions targeting technology use may be relevant in selected cases (Niu et al., 2023; Wang H. et al., 2024; Zhu et al., 2022). The need to differentiate ADHD presentations with and without internalizing comorbidity is also underscored. Interventions targeting family functioning and conflict resolution may be particularly critical when mood symptoms are present. Future research should prioritize longitudinal and multi-informant designs capable of modeling directional associations among ADHD symptomatology, medication status, IA, and specific relational dimensions over time, and should include samples with comorbid internalizing disorders to examine whether relational impairment is more pronounced and more globally distributed across dimensions in that subgroup. Examining ADHD presentation type as a potential moderator of relational outcomes, and collecting systematic data on parental mental health and family-level socioeconomic variables, would further strengthen the interpretability of future findings.

## 5 Conclusion

In conclusion, deficits in open communication are particularly evident among adolescents with ADHD who are not on medication, whereas adolescents receiving pharmacological treatment did not significantly differ from typically developing peers in open communication in this sample, though equivalence cannot be concluded in the absence of formal equivalence testing. IA showed a significant main effect on relational negativity across the full sample; whether this association varies by ADHD diagnosis or medication status was not formally tested and remains an open question for future research. These findings highlight the clinical relevance of communication patterns and digital-use behaviors in adolescents with ADHD, while longitudinal and multi-informant studies are needed before drawing stronger mechanistic or intervention-focused conclusions.

## Data Availability

The datasets analyzed during the current study are available from the corresponding author on reasonable request.
